# Frequency and Duration of Fecal Shedding of *Salmonella* Enteritidis by Experimentally Infected Laying Hens Housed in Enriched Colony Cages at Different Stocking Densities

**DOI:** 10.3389/fvets.2017.00047

**Published:** 2017-04-10

**Authors:** Richard K. Gast, Rupa Guraya, Deana R. Jones, Kenneth E. Anderson, Darrin M. Karcher

**Affiliations:** ^1^USDA Agricultural Research Service, U. S. National Poultry Research Center, Athens, GA, USA; ^2^Department of Poultry Science, North Carolina State University, Raleigh, NC, USA; ^3^Department of Animal Sciences, Purdue University, West Lafayette, IN, USA

**Keywords:** *Salmonella* Enteritidis, laying hens, conventional cages, enriched colony cages, stocking density, fecal shedding

## Abstract

Human infections with *Salmonella* Enteritidis are often attributed to the consumption of contaminated eggs, so the prevalence of this pathogen in egg-laying poultry is an important public health risk factor. Numerous and complex environmental influences on *Salmonella* persistence and transmission are exerted by management practices and housing facilities used in commercial egg production. In recent years, the animal welfare implications of poultry housing systems have guided the development of alternatives to traditional cage-based housing, but their food safety consequences are not yet fully understood. The present study assessed the effects of different bird stocking densities on the frequency and duration of fecal shedding of *S*. Enteritidis in groups of experimentally infected laying hens housed in colony cages enriched with perching and nesting areas. In two trials, groups of laying hens were distributed at two stocking densities (648 and 973 cm^2^/bird) into enriched colony cages and (along with a group housed in conventional cages at 648 cm^2^/bird) orally inoculated with doses of 1.0 × 10^8^ cfu of *S*. Enteritidis. At 10 weekly postinoculation intervals, samples of voided feces were collected from beneath each cage and cultured to detect *S*. Enteritidis. Fecal shedding of *S*. Enteritidis was detected for up to 10 weeks postinoculation by hens in all three housing treatment groups. The overall frequency of positive fecal cultures was significantly (*P* < 0.05) greater from conventional cages than from enriched colony cages (at the lower stocking density) for the total of all sampling dates (45.0 vs. 33.3%) and also for samples collected at 4–9 weeks postinfection. Likewise, the frequency of *S*. Enteritidis isolation from feces from conventional cages was significantly greater than from enriched colony cages (at the higher hen stocking density) for the sum of all samples (45.0 vs. 36.7%) and at 6 weeks postinoculation. Moreover, the frequency of *S*. Enteritidis fecal recovery from enriched colony cages at the higher hen stocking was significantly greater than from similar cages at the lower stocking density for all 10 sampling dates combined (39.4 vs. 33.3%). These results suggest that stocking density can affect *S*. Enteritidis intestinal colonization and fecal shedding in laying hens, but some other difference between conventional and enriched colony cage systems appears to exert an additional influence.

## Introduction

Although substantial government and agricultural industry resources have been invested in controlling food-borne diseases, the incidence of human *Salmonella* infections in the United States has not declined significantly over time (1, 2). Eggs contaminated by *Salmonella enterica* subspecies *enterica* serovar Enteritidis (*S*. Enteritidis) are internationally prominent sources of human illness (3, 4). Both active disease surveillance and retrospective epidemiologic analysis show an association between the frequency of human infections with this pathogen and its prevalence in commercial egg-laying poultry (5, 6). A survey of 24 European countries identified laying hens as the leading reservoir for human salmonellosis (especially due to *S*. Enteritidis), accounting for 42% of all cases (7). However, the sustained participation of egg producers in comprehensive *S*. Enteritidis flock testing and risk reduction programs (8) has recently been linked to decreased incidences of both egg contamination and human illness in several nations (9–11).

The edible interior contents of eggs (yolk or albumen) become contaminated with *S*. Enteritidis because this pathogen is able to colonize reproductive tissues (ovaries and oviducts) in infected hens (12, 13). Because salmonellae can be highly persistent in the environment of poultry houses, the opportunities for hens to be exposed and infected (and thus to lay contaminated eggs) can extend over a prolonged period of time (14, 15). Testing to detect *S*. Enteritidis in environmental samples from laying houses is often utilized as the initial screening step for identifying infected flocks (16, 17). Fecal shedding of S. Enteritidis by infected hens is a principal source of environmental contamination, often reaching peak levels just before egg production begins in commercial flocks and then declining steadily thereafter (18, 19). Experimental oral infection of chicks or hens with *S*. Enteritidis can cause intestinal colonization and associated bacterial shedding in feces for several months (20, 21).

The diverse available housing systems for commercial egg-laying hens have been extensively examined and evaluated in recent years in the contexts of their animal welfare and economic implications, but their public health consequences remain unresolved (22). Each of these housing options incorporates unique and complex facility characteristics and management practices, which might influence the persistence and transmission of *S*. Enteritidis infections in laying flocks. However, the published scientific literature does not provide any singular or definitive perspective about the food safety effects of poultry housing (23). Comparisons of conventional cage-based (battery) systems, cage-free systems, and intermediate alternatives such as enriched (furnished) colony cages or aviaries have yielded variable results, which do not document any consistent superiority of particular housing systems in the persistence of salmonellae in infected chickens or their housing environment (22). In a recent multi-institutional field study, the *Salmonella* prevalence in both environmental and eggshell samples was similar among several different hen housing systems, although unique inherent management challenges for sanitation and pathogen control were identified within each system (24). One characteristic parameter of poultry housing systems that might influence the introduction and perpetuation of *Salmonella* infections is the stocking density of hens (the amount of floor space available per bird). The objective of the present study was to determine the effects of two different bird stocking densities on the frequency and duration of fecal shedding of *S*. Enteritidis in groups of experimentally infected laying hens housed in colony cages enriched with perching and nesting areas.

## Materials and Methods

### Experimental Housing of Laying Hens

In each of 2 similar trials, 142 laying hens were obtained from the specific pathogen-free flock of Single Comb White Leghorn chickens maintained at the U. S. National Poultry Research Center in Athens, GA, USA. These hens (31- and 41-week-old at the beginning of the first and second trials, respectively) were distributed into three separately housed groups in different rooms of a disease-containment facility (biosafety level 2) containing cage systems designed to simulate commercial conditions. In one room, 42 hens were housed in conventional laying cages (6 hens per cage), which provided 648 cm^2^ of floor space per bird. Hens in the other two rooms were housed in enriched colony laying cages, each of which included access to two perches and a single enclosed nesting area. In one enriched colony room, 40 hens were housed (20 per cage) at a stocking density of 973 cm^2^ of floor space per bird. In the other enriched colony room, 60 hens were housed (30 per cage) at a stocking density of 648 cm^2^ of floor space per bird. All hens were provided with water (*via* two automatic nipple-type drinkers in each conventional cage and six in each enriched colony cage) and feed (a pelleted, antibiotic-free layer–breeder ration) *ad libitum*.

### Experimental Infection of Laying Hens with *S*. Enteritidis

In each trial, all hens were orally inoculated with a measured dose of *S*. Enteritidis, consisting of a mixture of strains of phage types 8 and 13a. Each *S*. Enteritidis strain was resuscitated by transfer into tryptic soy broth (Acumedia, Neogen Corp., Lansing, MI, USA) for two successive cycles of 24-h incubation at 37°C. After cell numbers in each incubated culture were estimated by determining their optical density at 600 nm, equal numbers of the two inoculum component strains were combined, and further serial 10-fold dilutions in 0.85% saline produced a final cell concentration in each oral dose of approximately 1.4 × 10^8^ cfu (confirmed by subsequent plate counts).

### Fecal Samples

Immediately before inoculation and at 10 weekly postinoculation intervals, sterile cotton swabs were used to collect samples of voided feces from polystyrene trays (food-grade but not sterile), which had been placed under each cage 1 day earlier. A total of 36 samples per room were collected on each sampling date, evenly distributed among all occupied cages (6 samples per conventional cage and 18 samples per colony cage). Feces selected for sampling were visibly moist (recently voided) and dark in color (characteristic of cecal discharge). Each sample was collected into 10 ml of buffered peptone water (Acumedia) and incubated for 24 h at 37°C. A 0.1-ml portion of each culture was then transferred into 10 ml of Rappaport-Vassiliadis broth (Acumedia) and incubated for 24 h at 41.5°C. A 10-µl portion from each of these broth cultures was then streaked onto brilliant green agar (Acumedia) supplemented with 0.02 mg/ml of novobiocin (Sigma Chemical Co., St. Louis, MO, USA) and incubated for 24 h at 37°C. The identity of presumptive colonies of *Salmonella* was confirmed biochemically and serologically (25).

### Statistical Analysis

Within each trial, between the two trials, and for both trials combined, significant differences (*P* < 0.05) between housing systems, hen stocking densities, or sampling dates in the mean frequencies of *S*. Enteritidis isolation from voided fecal samples were determined by Fisher’s exact test. Because the two replicate trials did not differ significantly in the frequency of *S*. Enteritidis recovery from fecal samples, their results were combined for analysis and presentation. Data were analyzed with InStat biostatistics software (GraphPad Software, San Diego, CA, USA).

## Results

None of the fecal samples collected before inoculation in either trial were positive for *Salmonella*. For both replicate trials combined, *S*. Enteritidis was recovered from 86.1% of fecal samples from hens in conventional cages, 95.8% of samples from hens in enriched colony cages at the higher stocking density, and 97.2% from hens in enriched colony cages at the lower stocking density at 1-week postinoculation (Table 1). The frequency of *S*. Enteritidis isolation from fecal samples collected in conventional cages declined significantly (*P* = 0.0014) to 59.7% at 3 weeks postinoculation, further to 40.3% at 5 weeks (*P* = 0.0344), and again to 19.4% by 9 weeks (*P* = 0.0112). In samples from enriched colony cages at the higher hen stocking density, *S*. Enteritidis recovery decreased significantly (*P* < 0.0001) to 62.5% at 3 weeks postinoculation, again to 27.8% at 5 weeks (*P* < 0.0001), and then to 12.5% by 8 weeks (*P* = 0.0378). For feces from enriched colony cages at the lower hen stocking density, the frequency of *S*. Enteritidis contamination dropped significantly (*P* = 0.0014) to 75.0% at 2 weeks postinoculation, again to 56.9% at 3 weeks (*P* = 0.0439), then to 36.1% at 4 weeks (*P* = 0.0219), to 18.1% at 5 weeks (*P* = 0.0251), and finally to 5.6% at 9 weeks (*P* = 0.0370). On the last sample collection date at 10 weeks postinoculation, *S*. Enteritidis was still found in 16.7% of fecal samples from hens in conventional cages, 8.3% of samples from hens in enriched colony cages at the higher stocking density, and 5.6% from hens in enriched colony cages at the lower stocking density.

**Table 1:** **Recovery of *Salmonella* Enteritidis^1^ from voided fecal samples of experimentally infected laying hens in different housing systems and stocking densities**.^2^

Weeks postinoculation	Conventional cages (high stocking density)	Enriched colony cages (high stocking density)	Enriched colony cages (low stocking density)

	*S*. Enteritidis-positive/total (%)		
1	62/72 (86.1)^a,A^	69/72 (95.8)^a,A^	70/72 (97.2)^a,A^
2	54/72 (75.0)^a,A,B^	62/72 (86.1)^a,A^	54/72 (75.0)^a,B^
3	43/72 (59.7)^a,B^	45/72 (62.5)^a,B^	41/72 (56.9)^a,C^
4	43/72 (59.7)^a,B^	34/72 (47.2)^a,b,B^	26/72 (36.1)^b,D^
5	29/72 (40.3)^a,C^	20/72 (27.8)^a,b,C^	13/72 (18.1)^b,E^
6	31/72 (43.1)^a,C,D^	17/72 (23.6)^b,C,D^	12/72 (16.7)^b,E,F^
7	23/72 (31.9)^a,C,D^	16/72 (22.2)^a,b,C,D,E^	11/72 (15.3)^b,E,F^
8	17/72 (23.6)^a,C,D^	9/72 (12.5)^a,b,D,E^	5/72 (6.9)^b,E,F^
9	14/72 (19.4)^a,D^	6/72 (8.3)^a,b,E^	4/72 (5.6)^b,F^
10	12/72 (16.7)^a,D^	6/72 (8.3)^a,E^	4/72 (5.6)^a,F^
All	328/720 (45.0)^a^	284/720 (39.4)^b^	240/720 (33.3)^c^

*^1^After oral inoculation of all hens with approximately 10^8^ cfu of an equal mixture of phage type 8 and 13a strains of S. Enteritidis*.

*^2^High stocking density = 648 cm^2^ of floor space per hen; low stocking density = 973 cm^2^ of floor space per hen*.

*^a,b^Values in rows that share no common lower-case superscripts are significantly (*P* < 0.05) different*.

*^A,B,C,D,E,F^Values in columns that share no common upper-case superscripts are significantly (*P* < 0.05) different*.

For both trials combined, the frequency of positive results for *S*. Enteritidis recovery from fecal samples was significantly greater for conventional cages than for enriched colony cages at the lower stocking density at 4 weeks (59.7 vs. 36.1%; *P* = 0.0089), 5 weeks (40.3 vs. 18.1%; *P* = 0.0061), 6 weeks (43.1 vs. 16.7%; *P* = 0.0011), 7 weeks (31.9 vs. 15.3%; *P* = 0.0313), 8 weeks (23.6 vs. 6.9%; *P* = 0.0099), and 9 weeks postinoculation (19.4 vs. 5.6%; *P* = 0.0217), as well as for the overall total of all 10 sampling dates (45.0 vs. 33.3%, *P* < 0.0001). The frequency of *S*. Enteritidis isolation from feces collected in conventional cages was significantly greater than from enriched colony cages at the higher hen stocking density at 6 weeks postinoculation (43.1 vs. 23.6%; *P* = 0.0228) and for the sum of all sampling dates (45.0 vs. 39.4%, *P* = 0.0219). The frequency of *S*. Enteritidis fecal recovery from enriched colony cages at the higher hen stocking was significantly greater than from enriched cages at the lower stocking density for the total of all 10 sampling dates (39.4 vs. 33.3%, *P* = 0.0185).

## Discussion

Fecal shedding is a consequence of *Salmonella* adherence to avian intestinal cells (26). Intestinal colonization by salmonellae typically declines steadily during the initial weeks after experimental infection of mature hens (21, 27), although highly persistent colonization has also been observed (18, 21). Following experimental oral infection with large doses of *S*. Enteritidis, a small percentage of hens housed in either conventional or enriched colony cages in the present study continued shedding the pathogen in their feces for at least 10 weeks. These results correspond with those of a prior study, which reported that inoculation with ≥10^6^ cfu of *Salmonella* led to fecal shedding for at least 8 weeks (21). Such prolonged shedding could extensively contaminate the housing environment and perpetuate opportunities for infection to spread. In some egg collection systems, contaminated feces can also introduce salmonellae onto egg shells. Nevertheless, interpolation from experimental infection data to predict housing and management influences on *Salmonella* shedding in commercial poultry must also account for some distinguishing characteristics of naturally occurring infections. The observed prevalence of *Salmonella* fecal shedding in commercial laying flocks sometimes fluctuates over time (28, 29). Both the frequency and duration of fecal shedding by orally infected hens are directly related to the *S*. Enteritidis exposure dose (21, 30, 31). Commercial laying hens are likely exposed to relatively low doses of salmonellae from environmental sources or *via* horizontal contact transmission, generally resulting in infrequent infection and egg contamination (9, 32).

Persistent environmental contamination in commercial poultry facilities serves as a potential reservoir for the infection of successive laying flocks with *S*. Enteritidis (8, 33, 34). Feces and dust, which are widely distributed throughout laying houses, sometimes remain contaminated with *S*. Enteritidis for many months (35). The ability of *S*. Enteritidis isolates to survive adverse environmental conditions may also correlate with their pathogenicity for chickens (36). High populations of rodent or insect vectors can sustain or amplify *Salmonella* levels in poultry flocks (37). The prevalence of *Salmonella* in laying house environments has been linked to several management-associated risk factors, including larger flock size, greater flock age, housing in older facilities, access to outdoor areas, and multiple-age stocking (38–41). Once introduced from environmental sources, *Salmonella* infection can rapidly and extensively spread within flocks (42). The susceptibility of chickens to horizontal transmission of *S*. Enteritidis can be increased by stressors such as feed deprivation, water deprivation, or exposure to extreme environmental temperatures (43–45).

Numerous and complex environmental influences on *Salmonella* persistence and transmission are exerted by management practices and housing facilities used in commercial egg production (46). However, prior investigations of the food safety consequences of poultry housing systems have yielded diverse and sometimes contradictory results (22, 23). For example, conventional cage-based housing systems for egg-laying flocks have sometimes been associated with higher frequencies of *Salmonella* infection or environmental contamination, especially when rodent population levels are elevated (41, 47, 48). Alternatively, other researchers have linked cage-free housing systems to higher *Salmonella* prevalence in egg shell and environmental samples and with greater horizontal dissemination of infection within laying flocks (49–51). Additionally, some studies have found no significant differences in the frequencies of either *Salmonella* infection or environmental contamination between cage and cage-free systems (52, 53) or between conventional and enriched colony cage systems (54, 55). Recently, a large field survey conducted under commercial egg production conditions found similar overall *Salmonella* prevalence in both egg shell and environmental samples from hens in conventional cage, enriched colony cage, and aviary housing systems, although salmonellae were isolated significantly more often from hens in conventional cages than from the other systems when internal organs were sampled after flock depopulation (24, 48). Unique *Salmonella* reservoirs and risk factors, attributable to the distinctive facility design features and management practices that are characteristic of individual poultry housing systems, may require correspondingly specific *Salmonella* risk reduction strategies for each system (24, 56).

In a series of previous experimental infection studies, *S*. Enteritidis was isolated significantly more often from internal organs and voided feces from hens in conventional cages than from hens in enriched colony cages, although no corresponding differences were reported for either horizontal transmission of infection or egg contamination (42, 57–59). Because the two housing systems in these trials differed in the amount of floor space provided per hen, a subsequent experiment (60) compared the effects of two stocking densities on the consequences of *S*. Enteritidis infection of hens in enriched colony cages. In this latter study, *S*. Enteritidis was found at higher frequencies in livers and ovaries of hens housed in enriched colony cages at a higher stocking density than at a lower one, but *S*. Enteritidis was also recovered at a higher frequency from spleens of hens in conventional cages than from enriched colony cages when both groups were housed at the higher density. In the present investigation, housing infected hens in enriched colony cages at a higher stocking density was associated with more frequent fecal shedding of *S*. Enteritidis than was detected at a lower stocking density, but hens in conventional cages (at the higher stocking density) shed the pathogen in their feces at a significantly higher overall frequency than either enriched colony cage group. These results suggest that the susceptibility of hens to intestinal colonization by *S*. Enteritidis can be influenced by stocking density, although some other characteristic of conventional cage housing appears to exert an additional effect.

Possible explanations for stocking density effects on the susceptibility of laying hens to *S*. Enteritidis infection include diminished immune responses or increased opportunities for horizontal contact exposure to the pathogen. Housing chickens in crowded and unsanitary conditions was previously reported to decrease their resistance to *S*. Enteritidis infection (61). Stress caused by high stocking densities has been found to suppress both humoral and cellular immunity, thereby facilitating increased invasion of internal organs by *S*. Enteritidis (62). Any disruptions of the complex regulatory circuitry, which coordinates immune responses, could reduce the effectiveness of host defenses against infection (63, 64). Stronger antibody and cellular immune responses were mounted by laying hens housed in enriched colony cages than by hens in conventional cages when subjected to social stress (65). Differences between housing systems and stocking densities in the frequency of colonization of the spleen (an important secondary lymphatic organ) in experimentally infected hens are consistent with a stress-mediated explanation (57, 60). Stress-related impairment of mucosal IgA secretion or other lymphocyte functions in intestinal lymphoid tissues could compromise effective clearance of *Salmonella* colonization (66, 67).

The carefully controlled conditions under which experimental infection studies are conducted are useful for evaluating the effects of narrowly defined treatments, but they cannot account for all of the complex management and environmental influences, which affect commercial egg production flocks and facilities. Accordingly, a comprehensive understanding of the public health impacts of different laying hen housing options can only be assembled by integrating experimentally derived data about housing system effects on hens’ susceptibility to *Salmonella* infection with applicable field data regarding the introduction, transmission, and persistence of this pathogen in commercial hens and their environment. Currently available information, encompassing both these research approaches, does not document any consistent overall pattern of significant differences between housing systems in their food safety consequences but instead suggests that effective control of egg-transmitted salmonellae may best be attained by addressing the specific risk factors inherent to each system.

## Ethics Statement

This study was carried out in accordance with the recommendations of the Institutional Animal Care and Use Committee and Institutional Biosafety Committee of the U. S. National Poultry Research Center and U.S. Department of Agriculture, Agricultural Research Service.

## Author Contributions

RKG was lead investigator and principal author; RG, DJ, KA, and DK were collaborators and coauthors.

## Conflict of Interest Statement

The authors declare that the research was conducted in the absence of any commercial or financial relationships that could be construed as a potential conflict of interest. The reviewer, CL (USDA-ARS, National Animal Disease Center), several of the authors (USDA-ARS, U. S. National Poultry Research Center), and handling editor (USDA-ARS, Southern Plains Agricultural Research Center) declared a shared affiliation, though no other collaboration and at different departments, and the handling editor ensured that the process nevertheless met the standards of a fair and objective review.
